# Analysis of Lrrn1 expression and its relationship to neuromeric boundaries during chick neural development

**DOI:** 10.1186/1749-8104-2-22

**Published:** 2007-10-31

**Authors:** Laura C Andreae, Daniela Peukert, Andrew Lumsden, Jonathan D Gilthorpe

**Affiliations:** 1 MRC Centre for Developmental Neurobiology, King's College London, New Hunt's House, Guy's Campus, London, UK, SE1 1UL; 2 Department of Neurophysiology, National Institute for Medical Research, The Ridgeway, Mill Hill, London, UK, NW7 1AA

## Abstract

**Background:**

The *Drosophila *leucine-rich repeat proteins Tartan (TRN) and Capricious (CAPS) mediate cell affinity differences during compartition of the wing imaginal disc. This study aims to identify and characterize the expression of a chick orthologue of TRN/CAPS and examine its potential function in relation to compartment boundaries in the vertebrate central nervous system.

**Results:**

We identified a complementary DNA clone encoding Leucine-rich repeat neuronal 1 (Lrrn1), a single-pass transmembrane protein with 12 extracellular leucine-rich repeats most closely related to TRN/CAPS. *Lrrn1 *is dynamically expressed during chick development, being initially localized to the neural plate and tube, where it is restricted to the ventricular layer. It becomes downregulated in boundaries following their formation. In the mid-diencephalon, *Lrrn1 *expression prefigures the position of the anterior boundary of the zona limitans intrathalamica (ZLI). It becomes progressively downregulated from the presumptive ZLI just before the onset of expression of the signalling molecule Sonic hedgehog (Shh) within the ZLI. In the hindbrain, downregulation at rhombomere boundaries correlates with the emergence of specialized boundary cell populations, in which it is subsequently reactivated. Immunocolocalization studies confirm that Lrrn1 protein is endocytosed from the plasma membrane and is a component of the endosomal system, being concentrated within the early endosomal compartment.

**Conclusion:**

Chick Lrrn1 is expressed in ventricular layer neuroepithelial cells and is downregulated at boundary regions, where neurogenesis is known to be delayed, or inhibited. The timing of *Lrrn1 *downregulation correlates closely with the activation of signaling molecule expression at these boundaries. This expression is consistent with the emergence of secondary organizer properties at boundaries and its endosomal localisation suggests that Lrrn1 may regulate the subcellular localisation of specific components of signalling or cell-cell recognition pathways in neuroepithelial cells.

## Background

Early neural development in vertebrates proceeds via the progressive regionalization of the neuroepithelium [1]. In some cases, most prominently the hindbrain and diencephalon, nascent regions become compartmented by differences in cell-cell affinity, which prevents the mixing of cells between adjacent regions. Cells lying at the boundaries between compartments may later form specialized signaling centres, or local (or secondary) organizers, that inform neigbouring cells about their position and fate. An important function of inter-compartment lineage restriction is that it stabilizes the position of the signal source and maintains a straight interface with the sink, both of which are crucial to the formation of consistent morphogen gradients.

Specialized local organizers pattern both the dorsoventral (DV) and the anteroposterior (AP) axes of the neural tube. The floor plate, at the ventral midline of the neural tube, has organizer activity through the ventralizing actions of Sonic hedgehog (Shh) and the transforming growth factor (TGF)β family member Nodal [2]. Similarly, at the dorsal midline, the roof plate exerts a dorsalizing activity by producing TGFβs and Wnts [3]. Along the AP axis, several boundaries have been characterized as local organizers. These include: the anterior neural border (ANB; also known as the anterior neural ridge in amniotes or Row-1 in zebrafish) at the anterior margin of the neural plate [4], which signals through secreted Wnt antagonists and the fibroblast growth factor (FGF)8 [5-7]; the zona limitans intrathalamica (ZLI), which signals through Shh and, perhaps, Wnt8b [8-10]; the midbrain-hindbrain boundary (MHB), which signals through the actions of FGF8 and Wnt1 [11]; and inter-rhombomere boundaries, which signal through Wnt1 [12,13].

In the chick embryo hindbrain, inter-rhombomere boundaries begin to form at Hamburger and Hamilton (HH) stage 9 [14] and develop into ridges or thickenings on the ventricular surface of the neural tube where the rate of mitosis is reduced. These ventricular ridges are associated with disrupted interkinetic nuclear migration [15], with cells being deflected into fan-shaped arrays on the apical-basal axis. Rhombomere boundary cells share a number of characteristics with radial glia, such as increased expression of the transcription factor *Pax6 *and the intermediate filament protein vimentin [16]. A similar phenomenon is observed at major boundaries in the forebrain, such as the diencephalic-mesencephalic boundary (DMB) and the prethalamic-thalamic boundary (ZLI) [17]. The conspicuous accumulation of axonal growth promoting extracellular matrix components highlights the subsequent function of some, but not all, boundaries as conduits for axon tracts [1,17-19].

Cells from neighbouring rhombomeres do not intermingle if the boundary between them is ablated microsurgically [20], or if boundary cell formation is blocked by the application of retinoic acid [21], indicating the lineage restriction between adjacent compartments is not imposed by a simple physical barrier to cell mixing, at least in the hindbrain. However, an early feature of rhombomere boundaries is a relative increase in the volume of extracellular space compared to rhombomere bodies, which is likely to be a consequence of the immiscibility between cells of neighboring rhombomeres due to cell-cell affinity differences [22-24]. Indeed, it has been known for some time that cells from alternate rhombomeres have differential affinities for one another [23], suggesting that this property could underlie the process of boundary formation. More recently, the molecular mechanisms responsible for this differential affinity have been identified following the observation that membrane associated Eph receptor tyrosine kinases and their ligands, the ephrins, have complementary expression in the odd and even numbered rhombomeres: boundary formation appears to be initiated by repulsive Eph-ephrin interactions at the interfaces between rhombomeres [24-26], while additional Eph-dependent adhesive mechanisms may act within rhombomeres to reinforce individual rhombomere integrity [27].

The mechanisms by which boundary cells themselves are specified are less clear, but are likely to involve signaling between rhombomeres. Boundary cells begin to express high levels of the glycosyl transferase radical fringe (Rfng), a likely regulator of Notch activation, whilst adjacent non-boundary cells express high levels of the Notch ligands DeltaA and DeltaD. Ectopic activation of the Notch pathway cause cells to segregate to the boundary. Moreover, inhibition of Notch signaling has an opposite effect, where cells sort away from the boundary. Thus, a high level of sustained Notch activation at the boundary appears to cause cells to adopt a boundary cell fate [28]. *Rfng *is also required for boundary cell expression of Wnt1, which regulates *Delta *and proneural gene expression in non-boundary zones, suppressing boundary cell markers [12,13]. Thus, boundary cells play a crucial role in coordinating the process of neurogenesis in the hindbrain, but their role in other brain regions is less well understood.

Boundary formation between dorsal (D) and ventral (V) compartments in the *Drosophila *wing imaginal disc shares a number of striking similarities with that in the vertebrate hindbrain. Early DV cell affinity differences, in concert with Notch signaling, establish the compartment boundary, which acts as a signaling centre through the action of wingless (Wg) to coordinate growth and patterning of the wing blade. D cell identity is specified by the LIM-homeodomain transcription factor apterous (*Ap*), which confers an affinity difference between D and V cells to restrict cell mixing at the DV boundary [29]. D cell affinity characteristics are conferred by Tartan (TRN) and Capricious (CAPS), two closely related type-I transmembrane leucine-rich repeat (LRR) proteins activated by *Ap *[30]. The establishment and maintenance of the affinity boundary appears to be only a transient function of Trn/Caps, which have been postulated to act as ligands for an unidentified cell surface receptor expressed on D cells [31].

We postulated that vertebrate LRR proteins might be involved in establishing compartment boundaries and identified the Leucine-rich repeat neuronal (Lrrn) family as being most closely related to TRN/CAPS based on sequence conservation. This three-member gene family consists of single-pass transmembrane proteins with conserved domain architecture.

Mouse Lrrn1 and Lrrn2 (first designated as neuronal leucine-rich repeat proteins (NLRR)-1 and -2, respectively) were the first members identified in a screen for LRR-containing proteins expressed in the mouse brain [32]. *Lrrn3 *was isolated shortly afterwards by the same group [33] and subsequently in rat [34]. Human *Lrrn2 *was identified independently as a gene that maps to chromosome 1 (*GAC-1*) and is overexpressed in gliomas, in which it is amplified [35]. More recently, the Lrrn nomenclature has become somewhat confusing. A mouse gene identified as *NLRR-4 *[36] encodes a distantly related protein (also known as C20orf75) that is not a member of the same family. Furthermore, a human gene identified as *hLRRN-5 *[37] belongs to a related but different family known as LINGO/LERN/LRRN6 [38,39]. However, the Human Gene Nomenclature Committee[40] has recently reinstated the name LRRN2 for LRRN5.

## Results

### Cloning, genomic structure and predicted protein sequence of chick Lrrn1

**Figure 1:**
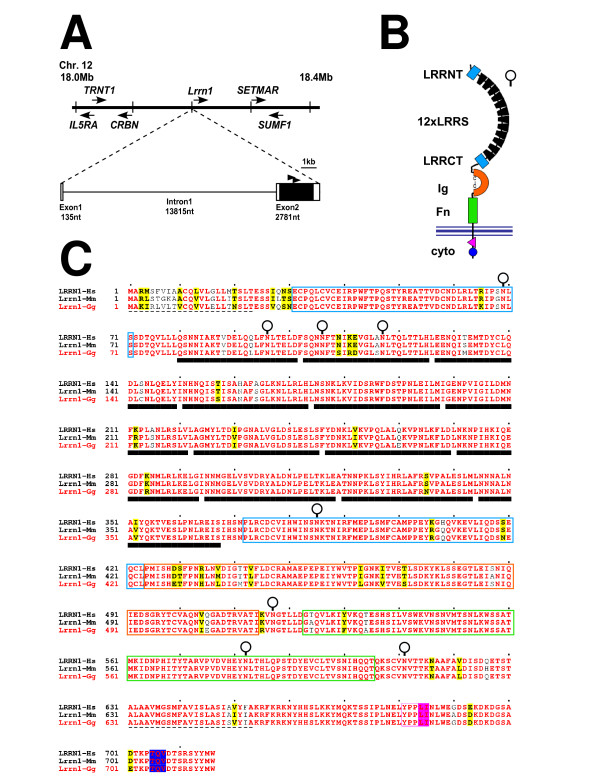
The chick *Lrrn1 *gene. **(a) **Map of the genomic interval containing *Lrrn1 *at approximately 18.2 Mb on chromosome 12 (horizontal black line). Vertical black lines represent 0.1 Mb intervals. Horizontal arrows represent the transcriptional orientation of genes identified within this interval and described in the Ensembl database (v.42). *Lrrn1 *is flanked by *cereblon *(*CRBN*) and a predicted gene (Ensembl: ENSGALG00000020777) related to human *SET domain and mariner transposase fusion gene *(*SETMAR*). Genomic organization of *Lrrn1 *predicted from the 2.9 kb cDNA clone identified is expanded below (scale bar = 1 kb). Predicted exons are shown as rectangles and the single intron as a black line. Sizes are shown as nucleotides (nt). The entire predicted coding region is contained on exon 2 (black shaded rectangle). Black arrowheads above exon 2 show the location of degenerate PCR primers (CDCVIHW, amino acids 375–381, 1,487–1,507 nt; and PEPEIYW, amino acids 453–459, 1,721–1,741 nt) used to amplify the *Lrrn1 *cDNA region used as a probe. **(b) **Schematic diagram of the Lrrn1 protein consisting of 12 extracellular LRRs (black isosceles trapezia) flanked by amino-terminal and carboxy-terminal flanking domains (blue rectangles), and single Ig domain (orange horseshoe, positions of conserved cysteine residues are indicated) and a membrane proximal FnIII domain (green rectangle). The plasma membrane lipid bilayer is represented by black horizontal lines. Locations of endocytic sorting motifs (pink triangle and blue circle) in the short cytoplasmic (cyto) tail are shown. **(c) **Predicted sequence of the chick (*Gallus gallus*) Lrrn1 protein (Lrrn1-Gg in red) aligned to its mouse (*Mus musculus*; Lrrn1-Mm; Ensembl: ENSMUSP00000037096) and human (*Homo sapiens*; Lrrn1-Hs; Ensembl: ENSP00000314901) orthologues (obtained from the Ensembl database) using the ClustalW method. Sequence positions are numbered on the left and marked at intervals of ten amino acids by black dots above. Identical residues amongst all three sequences are shown as red characters, similar residues are shown as black bold characters and boxed in yellow. Protein motifs are indicated using the same colour coding as in (b). Amino-terminal signal peptide and transmembrane domains are shown as dashed lines. Asparagine residues predicted to be potential sites of N-glycosylation are highlighted by an open circle.

To isolate chick *Lrrn1 *we used reverse transcriptase polymerase chain reaction (RT-PCR) to amplify part of the coding region (see Materials and methods). We then screened a phage λ cDNA library to obtain a 2.9 kb clone containing 5' and 3' untranslated regions (UTRs) and the entire predicted *Lrrn1 *open reading frame (ORF). Comparison of this sequence (GenBank: EF512462) with the Ensembl release of the chicken genome assembly (v.40, August 2006) confirms our identification. *Lrrn1 *maps to a position at 18.2 Mb of chromosome12 (Figure 1) in a region that is syntenic with human chromosome 3 and mouse chromosome 6. Importantly, our sequence improves on the gene prediction data provided by Ensembl for *Lrrn1 *(Ensembl: ENSGALG00000008279) by extending the 5' end to delimit the genomic position of the first and second exons. We do not know if the sequence of exon 1 is complete and extends as far as the 5' end, but our data fit with the known two-exon structure of *Lrrn1 *in human and mouse, whereby the entire ORF is contained on exon 2 and intron 1 is relatively large (15 kb in chick, 44 kb in human and 37 kb in mouse).

The deduced ORF of *Lrrn1 *encodes a protein of 716 amino acids that shows a remarkable degree of sequence identity to human and mouse (93%; Figure 1). Protein motif analysis reveals a domain structure characteristic of the Lrrn family. An amino-terminal signal sequence is followed by an array of 12 LRRs flanked by amino- and carboxy-terminal cysteine-rich flanking domains (LRRNT and LRRCT, respectively), a single immunoglobulin-like (Ig-like) domain and a fibronectin type III domain proximal to the single predicted transmembrane helix. The short intracellular domain (63 amino acids) contains only 2 amino acid substitutions between chick and human, both of which represent conservative changes. Within this region are two consensus endocytic sorting motifs (YXXΦ and di-leucine based) [41]. Lrrn1 also has a consensus internal class I post-synaptic density protein (PSD95), *Drosophila *disc large tumor suppressor (DlgA), and zo-1 protein (PDZ) ligand binding motif near the carboxyl terminus conforming to the consensus S/TxV/I/L [42,43]. The high degree of sequence conservation exhibited by Lrrn1 implies that it has maintained a strict degree of functional conservation during avian-mammalian divergence. Furthermore, it suggests that interactions with other proteins, binding partners and ligands have also remained conserved.

### Spatial and temporal expression of *Lrrn1 *mRNA

Chick *Lrrn1 *has previously been identified by two genetic screening approaches. First, as an unknown 3' expressed sequence tag (EST) clone 2B10 isolated from a subtracted hindbrain complementary DNA (cDNA) library and expressed in the forebrain, midbrain and r2/3 of the hindbrain at HH10+ [44]. Second, as a gene expressed in presomitc mesoderm, somites and neural tube at HH14–20 (clone 17-6G2) in a screen for signalling molecules expressed during somitogenesis [45]. During the preparation of this manuscript, a further characterisation of clone 2B10 in the central nervous system (CNS) was published, identifying it as *Lrrn1 *and detailing its temporal expression in the diencephalon [46].

**Figure 2:**
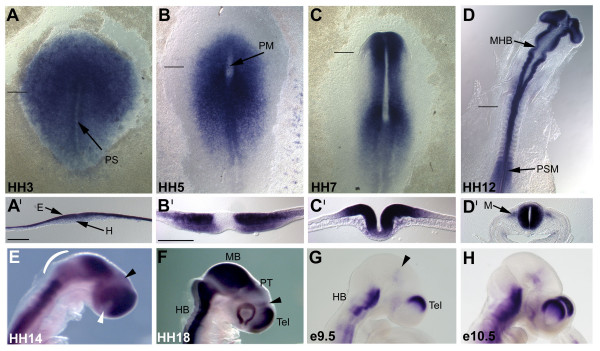
Spatiotemporal expression of *Lrrn1 *in the early embryo. *Lrrn1 *expression seen in whole-mount *in situ *hybridization specimens of **(a-f) **chick and **(g, h) **mouse embryos. **(a'-d') **Transverse vibratome sections of similar specimens to those in (a-d), respectively (plane and position of section indicated by a horizontal black line). Embryonic stage is indicated in the bottom left corner of each panel. (a) In the early chick, strong expression is seen throughout the neural plate in the epiblast layer (E in (a')) but not in the hypoblast (H) or primative streak (PS). (b) Downregulation in the prechordal mesendoderm of the head process (PM). (c) Strong expression in the neural plate but marked downregulation in the ventral midline. (d) Downregulation becomes evident at the mid-hindbrain boundary (MHB). Note the presence of expression in the unsegmented presomitic mesoderm (PSM) and in the myotomal component of the somites (M in (d')). (e) Downregulation in the isthmocerebellar region (white bracket), diencephalon and optic stalk (black and white arrowheads, respectively). (f) Strong expression in the hindbrain (HB), midbrain (MB), pretectum (PT), telencephalon (Tel) and at the prethalamus at the anterior margin of the ZLI (black arrowhead). Note the absence of expression posterior to this in the ZLI and dorsal thalamus. (g) Strong expression of mouse *Lrrn1 *in the hindbrain (HB) and dorsal telencephalon (Tel) at e9.5. Weak expression is seen in the pretectum (black arrowhead). (h) This pattern remains unchanged at e10.5. (a', d') 4×; (b', c') 10×. Scale bars in (a', b') = 200 μm.

In order to examine the temporal and spatial expression of *Lrrn1 *in the chick embryo, we performed a series of *in situ *hybridisations on HH3–18 embryos (see Materials and methods). We detected strong expression of *Lrrn1 *in the anterior epiblast of the primitive streak stage embryo, the earliest stage examined (HH3; Figure 2a,a'), while expression is weaker in the posterior epiblast and low, or absent, in the underlying hypoblast. At HH5 the prechordal mesendoderm of the head process extends anteriorly from Hensen's node and expression is downregulated in these regions (Figure 2b,b') and, notably, in the epiblast overlying the head process. *Lrrn1 *is strongly expressed in the neural plate and the underlying mesoderm lateral to the head process. As the neural plate begins to fold, expression becomes restricted to the basal and alar plate and to the neural folds, but remains absent from the midline – now the floor plate (Figure 2c,c'). Following closure, expression is seen throughout the neural tube but is downregulated strongly at the ventral and dorsal midlines (floor plate and roof plate, respectively; Figure 2d,d'), which is particularly evident in the hindbrain and midbrain. Thus, *Lrrn1 *appears to be selectively excluded from CNS midline signalling centres as they form. We also noted that *Lrrn1 *is expressed in the anterior presomitic mesoderm and myotome of the developing somites, as described previously [45].

From HH12 onwards, a further marked downregulation of expression is seen around the MHB (Figure 2d). By HH14, this has expanded to include the caudal midbrain and rostral hindbrain (r1) and downregulation is also seen in the mid-diencephalic territory (likely to correspond to the future thalamus, caudal to the ZLI [8,9]), and in the optic stalk (Figure 2e). This pattern is maintained through subsequent stages of development, but becomes more pronounced by HH18 (Figure 2f). Strong expression is maintained in the dorsal midbrain/pretectum and prethalamus, rostral to the ZLI, and telencephalon, but is downregulated at the telencephalic-diencephalic border (TDB). Whilst the striking absence of *Lrrn1 *expression from CNS boundary regions indicates a possible role in boundary formation or maintenance, the fact that it is also downregulated in other areas of the neuroepithelium, such as the isthmocerebellar region and the thalamus, may suggest alternative roles in proliferation or differentiation.

**Figure 3:**
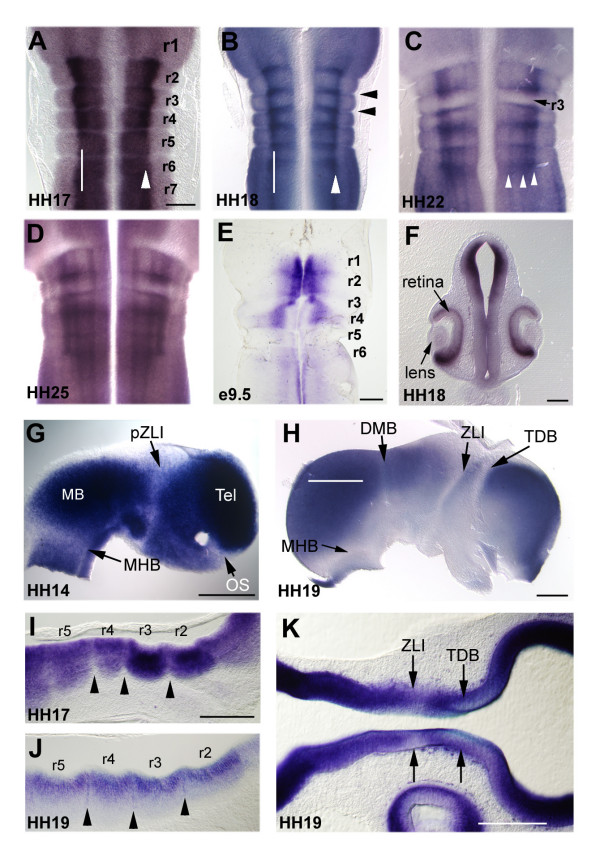
Relationship of *Lrrn1 *expression to neuromere boundaries. **(a-e, g-h) **Flatmounted or **(f, i-k) **vibratome sections showing *Lrrn1 *expression in the CNS of chick (a-d, f-k) and mouse (e) embryos. (a) Hindbrain preparation showing strong *Lrrn1 *expression restricted to a ventral domain with a clear dorsoventral boundary (white arrowhead). Note strong expression from rhombomere 2 (r2) caudally, but downregulation in rhombomere 1 (r1) and at interrhombomeric boundaries. (b) At HH18 downregulation has occurred in the core of the rhombomeres but strong expression is seen at boundaries, for example, at the r2/3 and r3/4 boundaries (black arrowheads) and in bilateral longitudinal stripes (white arrowheads). (c) At HH22 two further longitudinal stripes are evident medially (white arrowheads) and downregulation can be seen in r3 (black arrow). (d) A similar pattern persists at HH25. (e) Expression of mouse *Lrrn1 *in the hindbrain at e9.5 with a strong ventral distribution, bearing little relationship to that in the chick. (f) Frontal section of a HH18 chick embryo shows an additional site of expression in the ventral retina, but not in the lens primordium. **(g) **Hemisected, flatmounted preparation of the CNS with the eye removed and viewed from the ventricular surface. Strong *Lrrn1 *expression is evident in the midbrain (MB) and telencephalon (Tel). Downregulation can be seen at the mid-hindbrain boundary (MHB), in the thalamus at the presumptive ZLI (pZLI) and in the optic stalk (OS). **(h) **At HH19 downregulation has occurred throughout the basal plate of the forebrain and midbrain and is clearly distinguishable at principal neuromeric boundaries: telencephalic-diencepahlic boundary (TDB), mid-diencephalic boundary (ZLI), diencephalic-midbrain boundary (DMB), and the mid-hindbrain boundary (MHB). (i) Longitudinal section taken through the hindbrain at HH17 (white line in (a) marks plane of section). Positions of rhombomeres (r2–r5) are marked, confirming the existence of downregulation at interrhombomeric boundaries (black arrowheads). (j) Subsequent expression at the interface between rhombomeres can be seen at HH19 (black arrowheads). (k) Longitudinal section through the forebrain at the same stage (white line in (h) marks plane of section) showing downregulation at the TDB and ZLI. Scale bars = 200 μm.

Given the high degree of sequence conservation between chick and mouse Lrrn1, we also compared the expression of the mouse gene at comparable developmental stages (e9.5–10.5; Figure 2g,h). Somitic expression is conserved, as both mouse and chick *Lrrn1 *are upregulated at the lip of the dermomyotome as cells delaminate and migrate to form the myotome (data not shown) [47,48]). In the CNS, mouse *Lrrn1 *is expressed strongly in the dorsal telencephalon, albeit in a more restricted domain than in chick, and is virtually absent from the midbrain/pretectum (Figure 2f–h). Furthermore, the distribution of mouse *Lrrn1 *transcripts in the hindbrain is quite different (Figures 2g,h and 3e). Thus, the expression of mouse *Lrrn1 *does not show the same inverse relationship to boundary regions as does its chick counterpart and supports the notion that either its function is compensated for via alternative mechanisms, or it is not involved in a conserved mechanism underlying boundary formation.

To investigate the relationship between *Lrrn1 *expression and CNS boundaries in chick, we examined flat mounted preparations. We first examined expression in the hindbrain since this is an area of prominent segmentation where the mechanisms underlying the formation and development of rhombomere boundaries have been relatively well studied [12,16,18,21,24,28,49]. Between HH12–14, *Lrrn1 *occupies a ventrolateral territory either side of the floor plate that remains constant over this period (data not shown). At HH17, high-level *Lrrn1 *expression extending posteriorly from r2 into the spinal cord and is noticeably stronger at its dorsal most border (Figure 3a). Expression is stronger within the bodies of rhombomeres and weaker at inter-rhombomere boundaries (Figure 3a,i), which becomes discernable from HH14. However, from HH18 there is an inversion of this pattern: *Lrrn1 *becomes downregulated in the rhombomere bodies but is expressed strongly at boundaries (Figure 3b,j). It is also maintained along the dorsal-most border of its expression domain, which appears as a single longitudinal stripe extending posteriorly into the spinal cord (Figure 3b). At HH22, a striking downregulation of expression occurs within the whole of r3 (Figure 3c). By this stage, the pattern of longitudinal stripes has also diversified and is maintained until at least HH25 (Figure 3d). As seen in wholemount specimens, the pattern of *Lrrn1 *expression in the flat mounted mouse hindbrain is different from that in the chick. Mouse *Lrrn1 *is present in a domain immediately adjacent to the floorplate in r1–r3 and flares outwards into a more dorsal domain in r4 (Figure 3e). Expression is absent in r5 and r6. A coronal section through the head of a chick embryo at HH18 shows strong expression throughout the midbrain in the ventricular layer and in the ventral part of the retina (Figure 3f).

**Figure 4:**
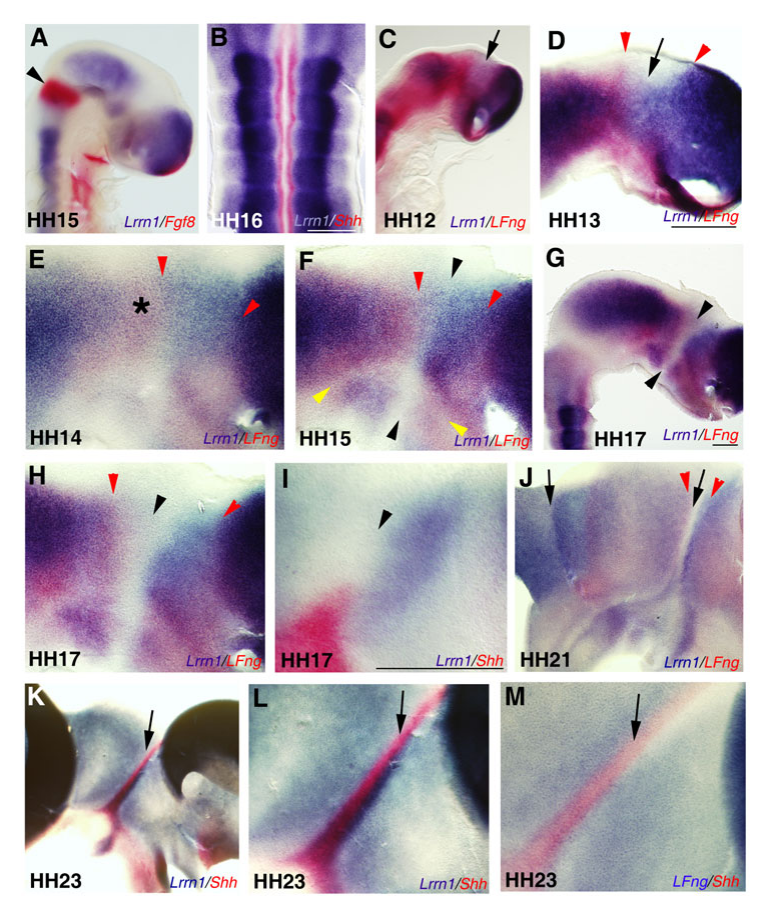
Dynamic expression of *Lrrn1 *in the diencepahlon. **(a) **Wholemount or **(b-m) **flatmounted specimens subject to double *in situ *hybridization for *Lrrn1 *(blue staining) and *Fgf8 *(a), *Shh *(b, i, k, l) or *Lfng *(c-h, j), or *Lfng/Shh *(m). Colour coded labels in the bottom right of each panel indicate the probes used. (a) *Lrrn1 *does not overlap with *Fgf8 *at the MHB (black arrowhead), or with *Shh *expression in the floorplate in (b). (c) Double *in situ *hybridization with *Lfng *shows downregulation in the prZLI region of the diencephalon (arrow) from HH12. (d) A hemisected, flatmounted preparation showing downregulation of *Lfng *and *Lrrn1 *in the diencephalic anlage. *Lrrn1 *is expressed within a wedge-shaped region in which *Lfng *not expressed (black arrow; boundaries of *Lfng *expression domains flanking the prZLI territory are marked by red arrowheads). (e) *Lrrn1 *expression clearly fills the *Lfng*-free wedge and abuts or partially overlaps the border of *Lfng *expression caudally. Note the low-level or absence of *Lrrn1 *expression in the thalamus itself (asterisk). (f) By HH15 the interface of the *Lrrn1/Lfng *domains has sharpened considerably to reveal the position of the ZLI (marked by black arrowheads) and the diencephalic basal-alar plate boundary (yellow arrowheads). (g) *Lrrn1 *downregulated in the ZLI at HH17. (h) Higher magnification view of the same specimen showing the ZLI as a *Lrrn1/Lfng*-free domain (black arrowhead). (i) Double *in situ *with *Lrrn1 *and *Shh *(red staining) showing strong domain of *Lrrn1 *expression immediately anterior to the ZLI (black arrowheads). (j) Strong expression on the anterior side of the ZLI compartment and the DMB at HH21 (black arrows). (k) At HH23 *Lrrn1 *abuts the expression domain of *Shh *on both sides of the ZLI (black arrow) as seen at higher magnification (l). It is expressed in the dorsal prethalamus, although *Lfng *is not (m). Scale bars = 200 μm.

The mechanisms underlying the formation of segmental boundaries in the midbrain and forebrain are less well understood than in the hindbrain. In order to compare the expression of *Lrrn1 *in the hindbrain with a more anterior region of the CNS, we hemisected and flat mounted specimens between HH12 and HH19, the period during which the ZLI becomes established as a developmental compartment in the mid-diencephalon [10,17]. High-level *Lrrn1 *expression was seen throughout the midbrain and forebrain at HH14. However, lack of expression was notable at the MHB, in the caudal part of the diencephalon (thalamus), in the optic stalk (Figure 3g) and at the anterior neural ridge (ANR, data not shown). By HH19, downregulation can be seen in basal areas throughout the entire midbrain and forebrain, but most strikingly at boundaries (Figure 3h,k). To confirm the position of *Lrrn1 *expression domains in relation to major boundaries, we performed a series of double *in situ *hybridisations. Lack of overlap between *Lrrn1 *and *fibroblast growth factor 8 *(*Fgf8*) at HH15 confirmed the exclusion of *Lrrn1 *from the caudal midbrain, MHB, r1 (Figure 4a) and ANR (data not shown). Similarly, non-overlap with *Shh *shows that *Lrrn1 *expression is not seen in the floorplate (Figure 4b). Cell-labelling studies have shown that the ZLI develops as a narrow compartment from a domain in which expression of *lunatic fringe *(*Lfng*) is excluded from HH12 onwards [10]. Double *in situ *hybridisation with probes for *Lrrn1/Lfng *show that *Lrrn1 *is also downregulated in the diencephalic primordium at around HH12 (Figure 4c,d). By HH14, the region devoid of *Lfng *expression extends more posteriorly than that of *Lrrn1 *(Figure 4e). The prethalamus and presumptive (pr)ZLI remain positive for *Lrrn1 *until HH16/17, but the thalamus is *Lrrn1*-negative. At HH15 a sharp *Lrrn1/Lfng *interface is visible at the position where the ZLI will form (Figure 4f), after which there is a gradual clearing of *Lrrn1 *within the prZLI. The ZLI forms in the alar region of the diencephalon and does not extend ventrally. However, *Lrrn1 *is also downregulated in the basal plate at the same anteroposterior position as the ZLI and at the basal/alar junction of the diencephalon (Figure 4f). The clearing of *Lrrn1 *from the prZLI at HH16/17 (Figure 4g,h) is intriguing since this marks the point where *Shh *first extends as a peak of expression from the floor plate of the diencephalon to occupy the core of the ZLI (Figure 4i) [10]. At later stages, *Lrrn1 *remains strongly expressed on the rostral side of the *Shh *expression domain in the ZLI, and on the rostral side of the diencephalic-midbrain boundary (Figure 4j,l). Whilst *Lfng *abuts the *Shh *domain on the caudal and rostral sides, it is not expressed dorsally in the prethalamus (Figure 4m). *Lrrn1*, however, abuts the *Shh *domain along its entire dorsoventral extent in the thalamus and prethalamus (Figure 4k,l). Thus, *Lrrn1 *is excluded from boundary regions of the rostral CNS and is downregulated in the ZLI compartment just before the appearance of *Shh*.

### Expression and localisation of Lrrn1 protein *in vivo*

**Figure 5:**
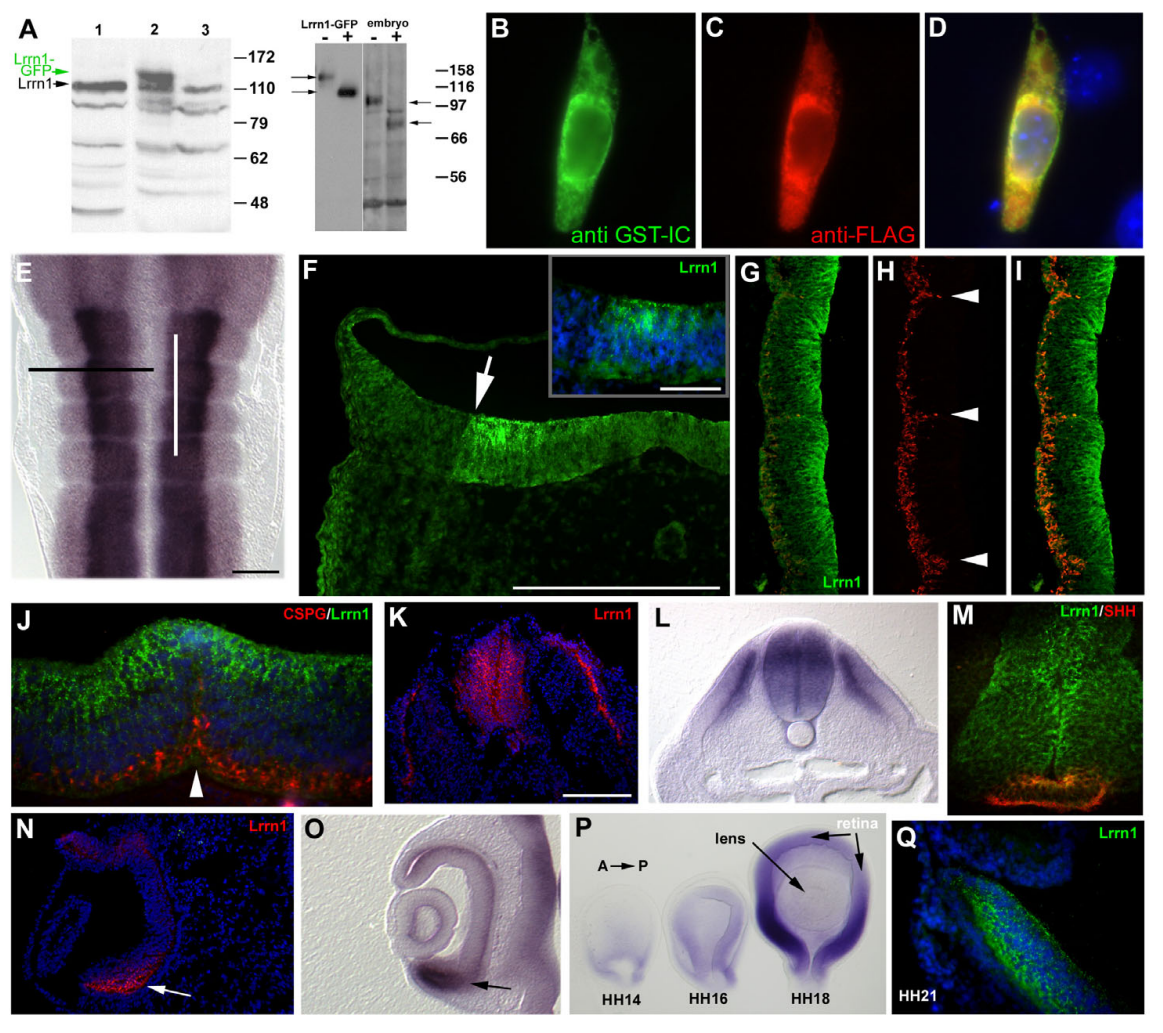
Distribution of the Lrrn1 protein. **(a) **Western blot analysis using the GST-IC antiserum. Left panel: whole-cell protein lysate from whole embryo (HH18, lane 1), or chick embryo fibroblasts (CEFs) transfected with a vector that expresses a Lrrn1-GFP fusion protein (lane 2), or empty vector control (lane 3). GST-IC recognizes a predominant species at 110 kDa (Lrrn1) also present in CEFs. The Lrrn1-GFP fusion protein runs at the expected size, approximately 27 kDa larger, at 140 kDa (green arrow). Right panel: control lysate (-) or lysate treated with PNGase F (+) from Lrrn1-GFP transfected Chinese hamster ovary (CHO) cells or HH18 embryos. In each case a shift of approx 30 kDa is seen after endoglycosidase treatment. Position of molecular weight protein standards is shown to the right in each case. **(b-d) **Epifluorescent images of a Neuro2a cell stained immunocytochemically following transfection (24 h) with a mouse FLAG-Lrrn1 construct and stained **(b) **with the GST-IC antiserum in green, **(c) **or an anti-FLAG antibody in red. **(d) **Merged image shows colocalisation of the two immunoreactivities (yellow). Nuclei are counterstained with Hoechst 33342 (blue). **(e) **Flatmount of HH17 hindbrain hybridised *in situ *for *Lrrn1 *as shown in Figure 3a. Black line indicates plane of section in (f). White line indicates plane of section in (g-i). **(f) **Lrrn1 immunohistochemistry with GST-IC shows strong staining in the ventricular layer of the hindbrain with a clear dorsoventral limit of expression (white arrow). Speckled, endosomal-like staining in NPCs in the ventricular region is shown at higher magnification (inset). Nuclei are counterstained with Hoechst 33342 (blue). **(g-i) **Longitudinal section through the hindbrain at HH17 stained with GST-IC (g) and the anti-neurofilament antibody RMO-270 (h), which labels axonal processes. Anterior is uppermost. (i) Merged image shows absence of colocalisation of the two markers, particularly at rhombomere boundaries (arrowheads in (h)). **(j) **Longitudinal section through the r4–5 boundary at HH18 double stained for Lrrn1 (green), chondroitin sulphate proteoglycan (CSPG using the CS-56 antibody, red) and counterstained with Hoechst 33342 (blue). Lrrn1 immunostaining is visible at the most apical region of the boundary (white arrow head) but not in more basal regions. **(k) **Cryostat section taken transversely through HH18 spinal chord stained for Lrrn1 (red). Hoechst 33342 counterstained nuclei are shown in blue. Note, congruence between Lrrn1 immunostaining and distribution of *Lrrn1 *RNA as shown by *in situ *hybridisation in a comparable section from a similar specimen **(l)**. Double labelling with Lrrn1 and SHH (5E1 antibody) shows that Lrrn1 is largely absent from the floorplate but some overlap exists in the apical region. **(n) **Cryostat section taken transversely through HH18 eye stained for Lrrn1 (red). Strong staining (white arrow) correlates strictly with strong staining for *Lrrn1 *by *in situ *from a similar specimen (**(o) **black arrow). **(p) **Optic cup/eye from HH14–18 specimens hybridised *in situ *for *Lrrn1 *(anterior to the right). Expression is seen in posterior, ventral-strong to anterior, dorsal-weak gradient. Transverse section through posterior, ventral part of the eye of a HH21 embryo stained for Lrrn1. Note, similar distribution of intensely labelled speckles in the retina as seen in other regions and absence of staining in the lens. Scale bars = 10 μm except in the inset of (f) = 5μm.

Lrrn family members are thought to be localized to the plasma membrane, where they could regulate cell adhesion or signalling [32,33,48,50]; however, *in vivo *evidence to support this hypothesis is lacking. To study the subcellular localisation of the Lrrn1 protein we raised an antiserum in rabbits to the intracellular domain of chick Lrrn1 using a glutathione S-transferase (GST) fusion protein incorporating the carboxy-terminal 57 amino acids (660–716) of the protein (GST-IC; see Materials and methods). Western blot analysis confirmed that the antiserum specifically recognizes a major polypeptide species migrating with an apparent molecular weight (Mr) of 110 kDa in HH18 embryo lysate (Figure 5a). Although Lrrn1 has a predicted Mr of approximately 80 kDa, the protein contains 8 potential N-linked glycosylation sites in the extracellular domain (Figure 1), which are strictly conserved in mouse. N-linked glycosylation occurs during synthesis of numerous transmembrane proteins in the rough endoplasmic reticulum and is important for protein conformation and stability and, hence, interaction with ligands or other factors. Treatment with the N-glycosidase PNGase F, followed by western blotting, was used to confirm the presence of N-linked glycosyl moieties (Figure 5a). PNGase F treatment of either HH18 embryo lysate or cells transfected with an expression construct encoding an Lrrn1-green fluorescent protein (GFP) fusion protein resulted in bands of the expected Mr for the fully deglycosylated protein (80 kDa for Lrrn1 and 107 kDa for Lrrn1-GFP). These results show that the chick protein is N-glycosylated *in vivo*.

The GST-IC antibody specifically detected a mouse Lrrn1-FLAG fusion protein in transfected cells (Figure 5b–d). We then confirmed the specificity of GST-IC *in vivo *by immunohistochemistry. We conducted our analyses on fresh-frozen cryosections subject to a short fixation protocol after sectioning (see Materials and methods). The distribution of staining obtained with GST-IC showed a strict congruence with the distribution of *Lrrn1 *mRNA by *in situ *hybridisation (Figure 5e,f,k,l,n,o). We did not observe any axonal labelling and staining in the hindbrain at HH17 as seen by lack of overlap with the neuronal neurofilament marker RMO-270 (Figure 5g–i). Staining was predominantly restricted to neural progenitor cells (NPCs) of the ventricular layer and was absent from rhombomere boundaries (Figure 5i–j). This indicates that, as at the RNA level, Lrrn1 is downregulated once cells leave the ventricular layer following differentiation and is absent from boundary regions. Interestingly, we noted that immunostaining within NPCs was strongest at the ventricular (apical) surface and in intensely labelled intracellular 'speckles' that have the appearance of endosomes in the hindbrain (Figure 5f). This is the first demonstration that Lrrn1 is a component of the endocytic compartment in NPCs and suggests that it may be involved in the endocytic regulation of cell adhesion or signalling mechanisms in these cells. The same distribution of staining was also seen at other sites of expression, for example, spinal cord (Figure 5k,m), retina (Figure 5n,q) and in the myotome (data not shown). We examined the temporal expression of *Lrrn1 *in the eye owing to the striking asymmetry in its distribution. Following the formation of the optic cup, expression becomes downregulated but remains detectable around the base of the cup where it joins the optic stalk at HH14 (Figure 5p). This domain has extended dorsalwards by HH16 but remains relatively weak and symmetrical along the AP axis. By HH18, however, expression has become upregulated strongly and exhibits an obvious ventral (strong) to dorsal (weak) gradient. Strongest expression is seen in the ventroposterior region of the retina and is absent from the lens (Figure 5n,o), which persists until at least HH21 (Figure 5q).

### Endosomal localisation of Lrrn1 protein

**Figure 6:**
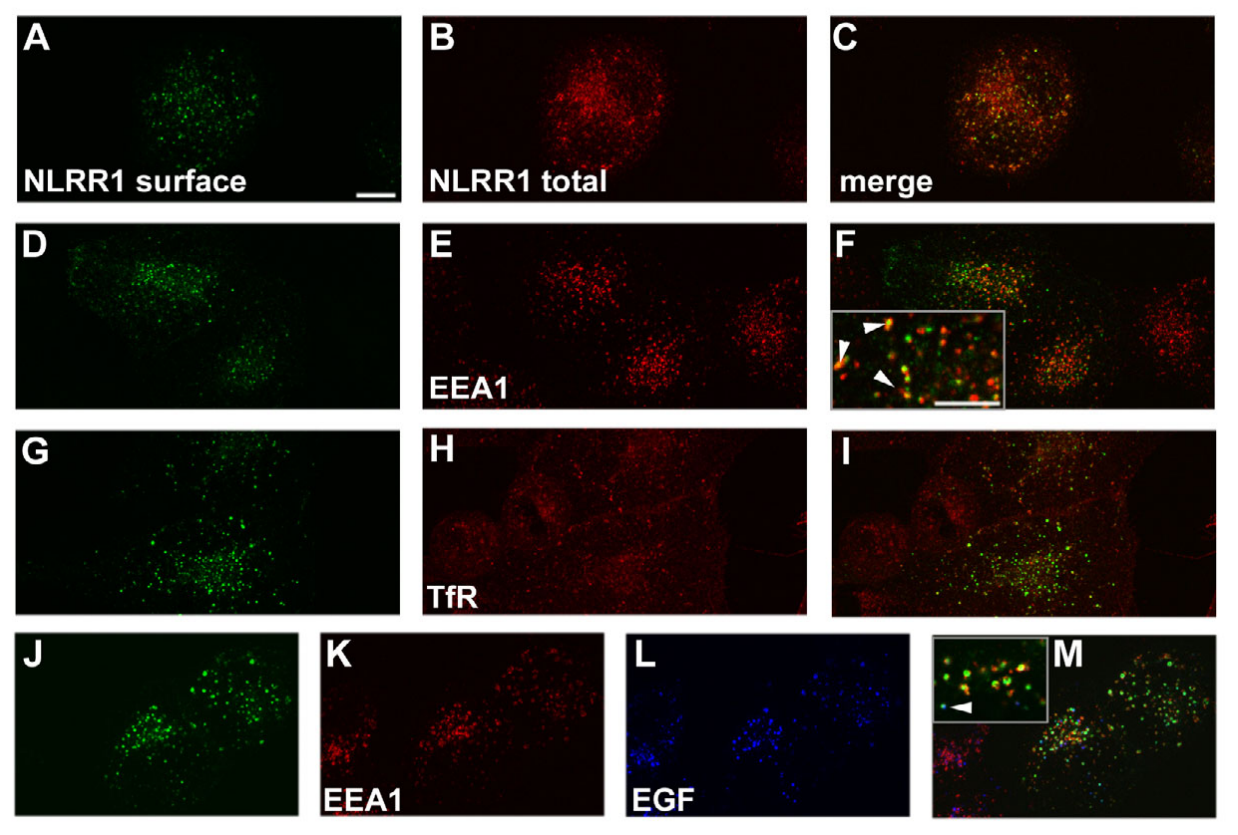
Endosomal distribution of Lrrn1. Immunocytochemical analysis of the endosomal uptake of FLAG-Lrrn1 in HeLa cells. **(a, d, g, j) **Plasma membrane surface-exposed FLAG-Lrrn1 protein detected with an anti-FLAG antibody. **(b, c) **Colocalisation with total cellular pool of Lrrn1 detected with the GST-IC antiserum. **(e, f) **Colocalisation with EEA1 (arrowheads in inset in (f)) shows that a significant pool of Lrrn1 cycles through the early endosomes. **(h, i) **Little overlap with transferrin receptor immunostaining suggests that Lrrn1 does not partition within recycling endosomes. **(k-m) **A very small proportion of endosomes were found to co-label with FLAG-Lrrn1/EGF ((m), arrowhead in inset). Scale bars = 200 μm except inset in (f) = 50μm.

To further characterize the endosomal localisation of Lrrn1, we performed live antibody labelling experiments on HeLa cells transfected with an amino-terminal FLAG-tagged mouse Lrrn1 construct (see Materials and methods). Cells were incubated with an anti-FLAG antibody in the culture medium to enable detection of protein present at the plasma membrane. Afterwards, cells were washed, fixed and permeabilized and the total pool of Lrrn1 detected using the GST-IC antibody. This enabled us to visualize the proportion of FLAG-Lrrn1 that was resident on the cell surface during the course of the experiment (30 minutes). Indeed, this approach shows that a relatively small proportion of the total cellular pool of Lrrn1 is exposed at the surface of the plasma membrane and is actively internalized into an endosomal compartment (Figure 6a–c). Colocalisation of internalized anti-FLAG with other markers enabled us to further assess the endosomal distribution of FLAG-Lrrn1. Significant overlap was seen with early endosomal antigen 1 (EEA1) immunostaining (Figure 6d–f). EEA1 is an established marker of early endosomes that functions as a cytoplasmic adapter for Rab5 to promote fusion [51]. EEA1 was found to decorate the periphery of many of the FLAG-Lrrn1^+ ^endosomes, indicating that the extracellular domain of Lrrn1 is exposed to the internal compartment of EEA1^+ ^endosomes. On the contrary, little or no colocalisation was seen between FLAG-Lrrn1 and the transferrin receptor (Tfr1/CD71; Figure 6g–i), which is a marker of recycling endosomes. We also examined colocalisation with fluorescently labelled epidermal growth factor (EGF), which, after 30 minutes, predominantly labels late endosomes/lysosomes in HeLa cells (JG, unpublished observation). Although some EGF^+^/FLAG-Lrrn1^+ ^vesicles were visible, we did not see a significant overlap with EGF. This may be the case with a shorter incubation period, but it was difficult to detect FLAG-Lrrn1 under these conditions.

## Discussion

### The Lrrn family

In mammals, the Lrrn family comprises three-members, *Lrrn1*, *Lrrn2/5 *and *Lrrn3 *[48]. Examination of the available genome data indicates that the same is true in avian species (JG, unpublished observation). The zebrafish genome contains two *Lrrn1 *genes. The first to be identified (*zfNLRR*) encodes a protein that is somewhat divergent from other Lrrn1s, being approximately 30 amino acids longer due to the presence of a number of insertions in the LRRCT-Ig-FnIII region [52]. A similar atypical *Lrrn1 *gene has recently been described in *Xenopus*, named *XlNLRR-6 *[53]. We have identified an additional *Lrrn1*-like gene in the zebrafish genome that is most similar to the other vertebrate *Lrrn1 *genes (Additional File 1 and Additional File 2). This points to the duplication of *Lrrn1 *early on in vertebrate evolution and the subsequent divergence of *zfNlrr/XlNLRR-6 *(which we refer to as *Lrrn1b*) from the archetypal *Lrrn1 *(*Lrrn1a*). The presence of *Lrrn1b *in both zebrafish and *Xenopus*, but not in mammals or birds, suggest that it has been lost in the amniote lineage, but to confirm this we must wait for more complete genome sequence data from representative reptilian and amphibian species.

Our identification and characterisation of a full-length cDNA clone for chick *Lrrn1 *shows that it encodes a 716 amino acid protein with a very high level of sequence identity to all vertebrate orthologues. This suggests that biochemical interactions between Lrrn1 and potential ligands/cofactors/binding partners are also likely to be highly conserved. Although comparison of the expression patterns of mouse and chicken *Lrrn1 *at comparable embryonic stages reveals some sites of coherence, such as the dorsal telencephalon and myotomal component of the somites, significant differences in other aspects of expression are evident between the two species. Mouse *Lrrn1 *does not bear the same characteristic relationship to neuromeric boundaries as chick *Lrrn1 *and is only weakly expressed in the midbrain and diencephalon. Neither *Lrrn2 *nor *Lrrn3 *are expressed in a way that could compensate for *Lrrn1 *in a redundant manner at neuromeric boundaries in mouse [48], suggesting that the functional role of Lrrn1 in relationship to boundaries is unique to birds, or that its function is compensated for in mammals by other factors. Although the function of Lrrn1 is currently unknown, several other families of single-pass transmembrane LRR-containing proteins have been identified (fibronectin leucine-rich transmembrane protein (FLRT)), leucine-rich transmembrane (LRRTM/LRTM) and LINGO/LRRN6/LERN (leucine-rich repeat neuronal) that could potentially fulfil this role [54-56]. All three share close structural relationships with the Lrrn family and their overall genomic organisation, whereby the coding region is almost exclusively contained on a single exon. Members of the *FLRT *family are FGF-responsive, encode binding partners for FGF receptors (FGFRs) and are likely to function as feedback modulators of FGF-MAP kinase signaling [57,58]. The LRRTMs form a four-member gene family widely expressed in the embryonic and adult mouse CNS [56,59]. The archetypal member of the LINGO/LRRN6/LERN family, LINGO-I, is a component of the Nogo66 receptor/p75 signalling complex [39]. The Lrrn and LINGO families share the same number of LRRs and each possesses a single Ig and FnIII domain. Although the embryonic expression of LINGOs in mouse has not been described in detail, chick LINGO-1 displays a number of overlapping and complementary sites of expression with Lrrn1 [60]. Careful comparison between the expression patterns of the Lrrn, LRRTM and LINGO family members in the CNS of mouse and chick should point to possible functional similarities between them.

### Dynamic expression of Lrrn1 in boundary regions

*Lrrn1 *is expressed throughout the neural plate of the chick embryo from its initiation, later becoming downregulated in a number of CNS regions that form boundaries. In the ventral midline, downregulation is coincident with floor plate formation following regression of Hensen's node from HH5/6 onwards. Similarly, in the dorsal midline (roof plate) downregulation is coincident with the onset of neural tube closure from HH6/7. The formation of both floor plate and roof plate is associated with the early differentiation of specialized cell types and the acquisition of signalling properties [2,3]. Thus, the loss of *Lrrn1 *from midline regions appears to correlate closely with the appearance of boundary properties and loss of neuronal precursor phenotype in them. At other boundaries, for example, in the mid-hindbrain, a temporal correlation does not hold. In chick, the MHB becomes morphologically distinguishable from HH8–9 whereas *Lrrn1 *downregulation becomes evident from HH12 and covers a domain that is considerably larger than the MHB itself, encompassing the caudal region of the midbrain, isthmus and r1.

### Hindbrain

*Lrrn1 *exhibits a biphasic expression pattern at rhombomere boundaries. In the first phase it becomes downregulated at boundaries and in the second, its expression is reactivated in the boundaries themselves. *Lrrn1 *downregulation at inter-rhombomeric boundaries becomes evident from HH14, several stages after their morphological appearance at HH9/10. Thus, it is unlikely that down-regulation plays a specific role in the initiation or establishment of boundaries in the mid-hindbrain region. One of the earliest detectable features of hindbrain boundaries is the local reduction in the rate of inter-kinetic nuclear migration, detectable from HH10 [15]. Furthermore, an increase in intercellular space in boundaries compared to non-boundary regions is also detectable from the same stage. The rate of increase remains constant until HH14, after which it becomes accelerated [18]. It is possible that *Lrrn1 *down-regulation is involved in this process of cellular rearrangement, since this is the stage when down-regulation in the basal plate of the hindbrain becomes evident. Although some markers have been characterized that are activated in boundary regions specifically around this stage, such as the *promyelocytic leukemia zinc finger *gene (*plzf*), *fgf3 *and *Pax6 *[16,61,62], further work is required to pinpoint its relationship to other genes that are involved in such a process. *Lrrn1 *upregulation in rhombomere boundaries between HH17 and HH18 coincides with the emergence of a morphologically and genetically distinct boundary cell population [16,18]. Thus, the dynamics of *Lrrn1 *expressions at boundaries may prove useful in understanding the cellular and genetic mechanisms involved in boundary maintenance and consolidation or the emergence of defined cell types with distinct boundary cell properties.

### Diencephalon

A similar pattern of downregulation followed by activation is also seen at the ZLI during a key stage of diencephalic development. The ZLI functions as a local organizer required for region-specific gene expression in the prethalamus and thalamus through the expression of Shh [8,9]. It forms a narrow compartment flanked anteriorly and posteriorly by linage restriction boundaries that bisects the diencephalon transversely [17]. The first indication of the position of the ZLI at earlier stages is the exclusion of *Lfng *from a wedge-shaped domain at HH12 [10]. *Lrrn1 *is also downregulated in the dorsal region of the *Lfng*-free prZLI at the same time and in the future thalamus, but expression remains in the prZLI until HH15. Expression then clears from the prZLI immediately prior to the intrusion of *Shh *into the ZLI from the basal region of the diencephalon. The timing of *Lrrn1 *down-regulation suggests that it may be involved in regulating the competence of this area of the neuroepithelium to express Shh. From HH17 onwards, *Lrrn1 *flanks the *Shh *domain at its anterior margin and remains highly expressed in this region. During the preparation of this work, another study describing the expression of chick *Lrrn1 *in the CNS was published suggesting that *Lrrn1 *prefigures the position of the ZLI before its overt formation [46]. Our results confirm and extend these findings by clearly showing, using double *in situ *hybridisation techniques, that *Lrrn1 *downregulation immediately precedes *Shh *expression and, thus, the emergence of organizer function.

### Correlation with the function of TRN and CAPS

The functional roles of TRN and CAPS in cellular recognition have been best characterized in the fly wing imaginal disc. Here they also demonstrate a biphasic pattern of expression with apparently distinct and separable functions. Early expression is restricted to the D compartment of the wing disc during the second instar. Early function seems to be to specify D cell affinity properties, which are important for the establishment of the DV boundary, requiring both extracellular and intracellular portions of the proteins [30,31]. During the third instar, expression becomes restricted to the lateral regions of the wing primordium and no longer respects the DV boundary. The later function for TRN/CAPS appears to be as cell survival factors and only requires the extracellular domain [31,63]. We have gone to great lengths to obtain functional evidence of a role for Lrrn1 in boundary formation/maintenance using gain of function (over expression) and loss of function (shRNA-mediated knockdown) approaches via *in ovo *electroporation. Neither of these has been successful in generating a discernable phenotype. This is not without precedent for LRR-containing proteins – the function of TRN/CAPS as specifiers of D cell affinity were only revealed via gain-of-function in a mutant background where normal expression had been removed. Loss-of-function experiments using TRN/CAPS double mutant clones also did not have an overt effect on the patterning of the wing. Hence, it is possible that we will need to generate suitable loss-of-function constructs to interfere with Lrrn1 effectively.

Work from Stephen Cohen's group has predicted the existence of an as-yet uncharacterized cellular receptor for TRN/CAPS and found no evidence of homophilic interactions [30,31,63]. However, a recent study examining the function of the same proteins in the leg imaginal disc suggests that CAPS, and perhaps TRN also, act homophilically as cell-cell recognition molecules to mediate boundary refinement and induce invasive cellular behaviour [64]. TRN/CAPS show limited but significant sequence similarity to chick Lrrn1 in their extracellular regions (26% across the LRRNT-LRR-LRRCT portions of the proteins, data not shown) and a short region of identity within the membrane proximal intracellular domain ('QKT' motif; Additional file 3). Although no evidence exists that points to TRN/CAPS and Lrrn1 being functional homologues, a hypomorphic mutant of CAPS has been identified that carries a point mutation (T501I), which is conserved between the three proteins and may prove useful in directing the design of loss-of-function tools for Lrrn1. Furthermore, TRN/CAPS also show a similar distribution of punctate cellular staining in the leg disc as we have seen for Lrrn1 in chick. We have adopted strategies to identify a ligand or co-receptor for Lrrn1 that have been widely employed to identify interacting partners for similar proteins. We have screened more than 5 × 10^5 ^clones of an embryonic mouse cDNA expression library [65] using the extracellular portion of Lrrn1 fused to alkaline phosphatase as a probe. We have also used the same reagent to look for binding to wholemount embryos and fresh-frozen cryostat sections under a wide range of binding conditions. Neither approach has proved successful. Furthermore, we do not see aggregation of Lrrn1 transfected cells in a manner that suggests homophilic cell adhesion, as has been seen for other LRR proteins such as CAPS [66] or FLRT3 [67]. Thus, our evidence, albeit negative, strongly points to the fact that Lrrn1 does not interact homophilically. Nor does it appear to simply bind another ligand or receptor in *trans*. Thus, it is likely either to form a multiprotein receptor complex with other coreceptors or to bind to a complex or small molecule ligand.

## Conclusion

The conserved nature of Lrrn1 suggests that it plays an important function during CNS development. It is dynamically expressed throughout the neuroepithelium but is restricted to NPCs and broadly downregulated in neuromere compartment boundaries. The endosomal localisation of Lrrn1 suggests that it may be involved in the regulation of signalling in progenitor cells. By analogy to related proteins, it has the potential to scaffold (via a PDZ-domain binding site) and regulate the subcellular trafficking of groups of functionally interacting molecules, such as receptors or ion channels, and could thereby regulate the integration of signalling pathways in NPCs.

## Materials and methods

### Cloning of chick *Lrrn1 *and sequence analysis

Degenerate primers were designed to conserved amino acids of human (GenBank: AAQ88679), mouse (GenBank: NP_032542) and *Xenopus *(GenBank: AAH59292) Lrrn1. These corresponded to the peptide sequences CDCVIHW (amino acids 375–381: 5'-TGYGAYTGTGTMMTSCRYTGG-3') and PEPEIYW (amino acids 453–459: 5'-CCHSARCCMGARATHTACTGG-3'; Figure 1). Primers were used to amplify HH10–12 chick embryo cDNA (a gift of Dr D Chambers) with a 3' *Not*I-(dT)_17 _primer. The resulting 1,689 base-pair (bp) fragment encompassing the carboxy-terminal coding region (amino acids 453–717) and 3'-UTR (872 bp) of *Lrrn1*, was cloned into pCR4^®^TOPO^® ^(Invitrogen. Paisley, Renfrewshire, UK) and verified by sequencing (pCR4TOPO-cLrrn1). A 575 bp *Sac*I-*Ssp*I fragment of the *Lrrn1 *cDNA was then used to generate a radioactively labelled probe with [α-^32^P]dCTP. Screening a λZAPII cDNA library (gift of Prof. D Wilkinson) resulted in the identification of a 2.9 kb full-length clone, which was verified by DNA sequencing (GenBank accession number EF512462). Multiple sequence alignments were performed using ClustalW [68] using default parameters and annotated using ESPript [69]. Protein motifs were identified using the InterProScan suite of programs [70] or Scansite [71].

### *In situ *hybridisation

Chick (Rhode Island Red) or mouse (CD11) embryos were fixed overnight at 4°C in 4% (w/v) paraformaldehyde (PFA) in phosphate buffered saline (PBS; pH7.2). Chick embryos were staged according to Hamburger and Hamilton (HH) [14]. *In situ *hybridisation was performed as described previously [72]. pCR4TOPO-cLrrn1 was linearized with *Pvu*II and transcribed with T3 RNA polymerase (Roche, Burgess Hill, West Sussex, UK) to generate an antisense digoxigenin (DIG)-uracil triphosphate (UTP)-labelled riboprobe (DIG RNA Labeling Mix, Roche). A sense probe, transcribed with T7 RNA polymerase after linearization with *Spe*I, gave no specific signal (data not shown). For double *in situ *hybridisation, the second probe was labelled with fluorescein isothiocyanate (FITC)-dUTP (FITC RNA Labeling Mix, Roche) and both DIG/FITC probes (1 ng/ml each) were hybridized concurrently. After completion of the detection step for DIG with NBT/BCIP as a substrate (NBT/BCIP Stock Solution, Roche; 9.4 μg/ml nitroblue tetrazolium chloride and 4.7μg/ml 5-bromo-4-chloro-3-indolyl-phosphate), alkaline phosphatase (AP) conjugated sheep anti-DIG Fab antibody (Roche) was inactivated by washing embryos (3 × 5 minutes) in 0.1 M glycine-HCl (pH 2.2). The FITC detection step was then performed using sheep anti-FITC-AP (Roche) and Vector^® ^Red substrate (Vector Laboratories, Burlingame, CA, USA). Additional plasmid templates were used to generate antisense riboprobes as follows: mouse *Lrrn1 *(*Hin*dIII-T7) [48]; chick *Shh *(*Sal*I-SP6; gift of T Lints); chick *Fgf8 *(*EcoR*1-T7; gift of G Martin); chick *lunatic fringe *(*Cla*I-T3; gift of C Tabin).

Embryos were refixed overnight in PFA and cleared in 87% (v/v) glycerol prior to digital photography of wholemounts (Olympus DP70 attached to a stereo dissecting microscope). Some specimens were dissected and flatmounted under a No. 1.5 coverslip in glycerol. Vibratome (Leica VT 1000S) sections were cut at 40–50 μm after embedding tissue in 20% (w/v) gelatine/PBS. Following infiltration in gelatine at 65°C for 1–2 h, tissue was embedded and post-fixed in PFA containing 0.1% (w/v) glutaraldehyde. Flatmounts and sections were photographed on a Zeiss Axiophot microscope equipped with a Zeiss Axiocam or Olympus DP70 CCD camera.

### Generation of anti-Lrrn1 antisera

A fragment corresponding to the intracellular domain of the chick Lrrn1 (IC, amino acids 660–716), including the translational termination codon, was generated by PCR with primers 5'-ACGGATCCAAAACTACCACCATTCAC-3' and 5'-CGCCTCGAGTTACCACATGTAATAGC-3', cloned into the pCR4^®^TOPO^® ^vector (Invitrogen) and verified by sequencing. IC was fused in frame to GST) by cloning a *Bam*HI-*Xho*I fragment (restriction sites underlined above) into pGEX-5X-3 (GE Healthcare, Little Chalfont, Buckinghamshire, UK). The GST-IC fusion protein was expressed in *Escerichia coli *(BL21 strain, Promega, Southampton, Hampshire, UK) and purified on a glutathione sepharose 4B column (Microspin, GE Healthcare) following lysis and mild sonication. Polyclonal antisera against the purified GST-IC protein were raised in rabbits (Eurogentec, Seraing, Belgium).

### Western blotting and PNGase F treatment

Embryo or cell extracts were made by homgenization (via pipetting) and lysis in ice-cold RIPA buffer containing a cocktail of protease inhibitors (Complete, Roche). Lysates were extracted for 1–2 h at 4°C with gentle rocking followed by microcentrifugation (13,000 × g at 4°C for 20 minutes). Cleared lysates were stored at -20°C. Western blotting was performed with supported nitrocellulose membranes using standard techniques following denaturing SDS-PAGE on an 8% polyacrylamide gel (Mini-Protean III system. Bio-Rad, Hemel Hempstead, Hertfordshire, UK). The GST-IC antibody was used at a dilution of 1:6,000 and detected using a horseradish peroxidase-coupled secondary antibody (Vector Laboratories, 1:1,000) followed by an enhanced chemiluminescent detection system (Pierce, Rockford, IL, USA). PNGase F (New England Biolabs, Hitchin, Hertfordshire, UK) digestion was performed on 15 μl (approximately 20 μg) of HH18 embryo lysate or 5 μl (approximately 5 μg) of Lrrn1-GFP transfected Chinese hamster ovary (CHO) cell lysate according to the manufacturers instructions, using a total of 2 μl (1,000 U) of PNGase F (2 h at 37°C).

### Immunohistochemistry

Unfixed chick embryos were directly embedded in OCT compound (Sakura Finetech, Zoeterwoude, (The Netherlands) on dry ice and stored at -80°C. Frozen sections (8–20 μm) were cut on a Bright OTF cryostat at -15°C and dried briefly (30 s) on a hotplate at 55°C and then fixed for 10 minutes at room temperature using PFA. Longer fixation protocols resulted in a dramatic reduction in signal:noise. Sections were washed in TBST (100 mM Tris pH 7.5, 150 mM NaCl, 0.1% (v/v) TritonX-100), blocked with 10% (v/v) foetal calf serum (FCS) in TBST for 1 h at room temperature and incubated in primary antibody (in block) overnight at 4°C. Sections were washed in TBST and incubated in fluorescently conjugated goat secondary antibodies (Alexa Fluor^®^, Invitrogen) diluted 1:1–2,000 in TBST/1% (v/v) FCS for 1 h at room temperature. After washing in TBST, sections were coverslipped in a glycerol-based mounting medium (Citifluor, London, UK). Nuclei were conterstained with Hoechst 33342 (0.3μg/ml, Invitrogen) by inclusion in the final wash step. Sections were photographed on a Nikon Eclipse microscope using a Zeiss Axiocam.

### Cell transfection and immunocytochemistry

Mouse neuroblastoma (Neuro2a) or human HeLa cells were seeded on sterile 13 mm diameter glass coverslips (No. 1.5) in DMEM supplemented with 10% (v/v) FCS, 2 mM L-Alanyl-L-Glutamine (GlutaMAX™I, Invitrogen), 1,000 u/ml penicillin, 1,000μg/ml streptomycin and 25μg/ml of amphotericin B (Antibiotic-Antimycotic, Invitrogen) in a humidified 37°C incubator/8% (v/v) CO_2_. Cells were transfected at 30–50% confluence with either a carboxy-terminal [48] or amino-terminal (gift of Dr Bryan Haines) FLAG-tagged mouse *Lrrn1 *expression vector. For live labelling experiments, N-FLAG-Lrrn1 expressing cells were incubated with mouse anti-FLAG^® ^M2 monoclonal antibody (1:500, Sigma, Gillingham, Dorset, UK), or also in the presence of Alexa^® ^Fluor 633-labelled EGF (10μg/ml, Invitrogen), for 30 minutes at 37°C. They were washed by transferring between 4 wells containing 500μl of warm culture media and fixed by adding 500μl 8% (w/v) PFA to the final wash step. Cells were washed in PBS and permeabilized with 0.3% (v/v) TritonX-100/PBS for 10 minutes. Total Lrrn1 protein was detected with the IC antibody (1:1,500) followed by an Alexa^® ^Fluor 488 goat anti-rabbit secondary antibody (1:2,000, Invitrogen). The antibody against EEA1 (rabbit polyclonal antiserum 1:1,500) was a kind gift of Dr Mario Zerial (EMBL, Heidelberg, Germany) and the anti-human transferrin receptor (rabbit polyclonal, 1:250) was from Chemicon. Millipore, Watford, Herfordshire, UK) Internalized anti-FLAG was detected with an Alexa^® ^Fluor 568 goat anti-mouse secondary antibody. Cells were mounted in Citifluor and imaged on a Zeiss LSM 510 confocal microscope using 63× (NA 1.3) oil immersion objective.

## Competing interests

The author(s) declare that they have no competing interests.

## Authors' contributions

LA and JG carried out the molecular, genetic and biochemical studies. LA compiled the figures and JG performed the sequence alignment, drafting and compilation of the manuscript. AL played an integral part in the conception of the study, and participated in its design and coordination and helped to draft the manuscript. DP conducted molecular, genetic and biochemical studies during the revision of the manuscript. All authors read and approved the final manuscript.

## Supplementary Material

Additional file 1 Comparison of vertebrate Lrrn1 proteins. Protein sequence alignments of vertebrate Lrrn1 proteins. Click here for file

Additional file 3 Conserved motif in the intracellular domain of tartan, capricious and Lrrn1. Protein sequence alignment of conserved motif in the intracellular domain of tartan, capricious and Lrrn1. Click here for file

Additional file 2 Comparison of chick and zebrafish Lrrn1 proteins. Protein sequence alignment of chick and zebrafish Lrrn1 proteins. Click here for file
