# Supplementary material for: Analysis of Lrrn1 expression and its relationship to neuromeric boundaries during chick neural development
Source: Neural Dev. 2007 Oct 31;2:22. doi: 10.1186/1749-8104-2-22 (PMC2225406; doi:10.1186/1749-8104-2-22)
Supplement: Additional file 1 — Comparison of vertebrate Lrrn1 proteins. Protein sequence alignments of vertebrate Lrrn1 proteins. [file 1749-8104-2-22-S1.pdf]

Additional file 1: Comparison of vertebrate Lrrn1 proteins

A

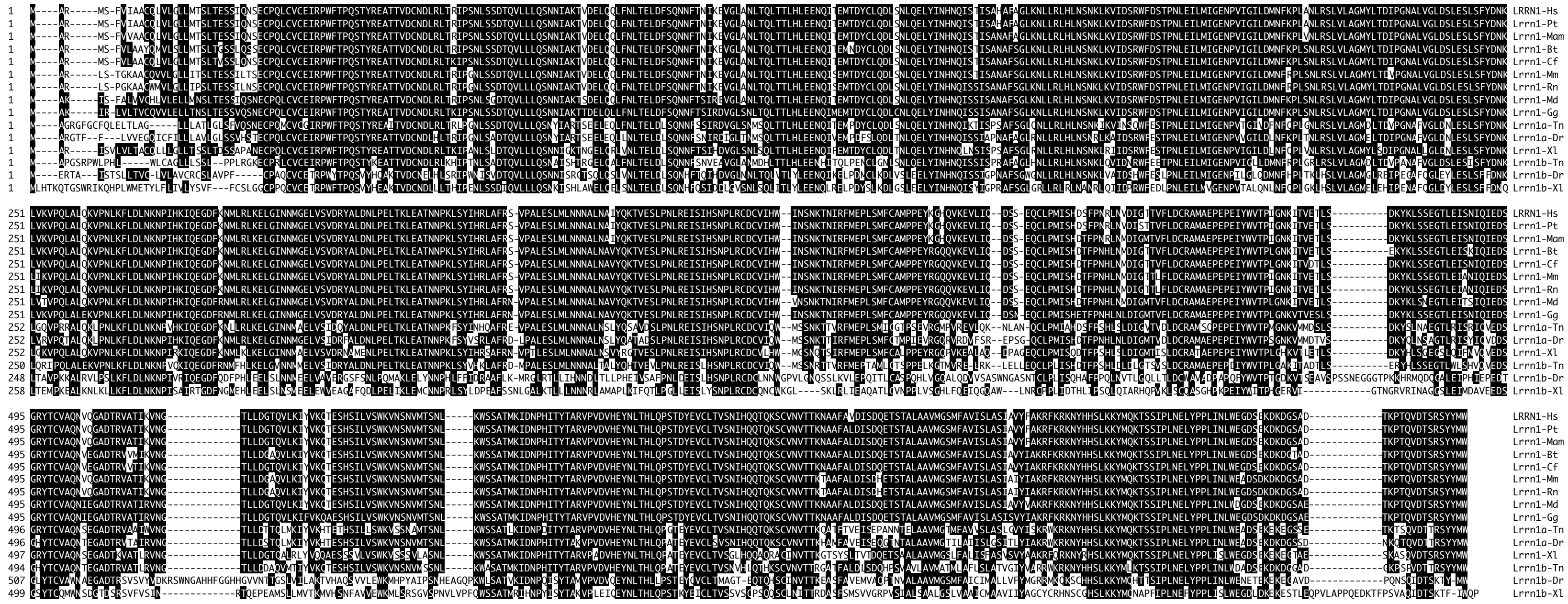

B

Divergence

	1	2	3	4	5	6	7	8	9	10	11	12	13	14	15		
1		99.7	99.0	96.2	97.3	95.5	95.5	94.3	92.7	77.5	79.9	79.2	74.5	50.6	46.5	1	LRRN1-Hs
2	0.3		99.0	96.2	97.3	95.5	95.5	94.3	92.7	77.2	79.6	79.2	74.5	50.3	46.5	2	Lrrn1-Pt
3	1.0	1.0		96.6	97.8	95.9	95.9	95.0	93.3	77.2	80.3	79.7	74.7	50.4	46.5	3	Lrrn1-Mam
4	3.9	3.9	3.4		98.2	95.5	95.5	94.8	93.4	77.2	80.4	79.5	75.0	50.7	47.1	4	Lrrn1-Bt
5	2.7	2.7	2.3	1.8		96.1	96.1	95.4	94.3	77.8	80.7	80.4	75.2	50.6	46.8	5	Lrrn1-Cf
6	4.6	4.6	4.2	4.6	4.0		98.9	93.4	92.6	77.1	79.5	79.1	74.5	50.1	46.6	6	Lrrn1-Mm
7	4.6	4.6	4.2	4.6	4.0	1.1		93.9	92.7	76.7	79.5	79.3	74.7	50.3	46.9	7	Lrrn1-Rn
8	6.0	6.0	5.2	5.4	4.8	6.9	6.4		95.4	77.8	80.6	80.4	75.0	51.0	46.6	8	Lrrn1-Md
9	7.7	7.7	7.0	6.9	6.0	7.8	7.7	4.8		77.5	80.2	81.6	74.0	50.8	47.2	9	Lrrn1-Gg
10	25.9	26.3	26.3	25.9	25.5	26.3	26.9	25.5	25.5		81.3	72.8	71.3	48.8	45.9	10	Lrrn1a-Tn
11	22.6	23.0	22.1	22.1	21.7	23.0	23.0	22.1	22.4	20.2		73.4	73.7	49.9	46.0	11	Lrrn1a-Dr
12	23.3	23.3	22.5	22.9	21.6	23.5	23.1	21.6	20.1	32.6	31.8		69.9	50.4	47.8	12	Lrrn1-Xl
13	28.8	28.8	28.6	28.4	28.0	28.6	28.6	28.4	29.8	34.7	31.0	36.4		48.3	45.6	13	Lrrn1b-Tn
14	64.0	64.7	64.3	63.7	63.1	65.0	64.3	62.8	62.8	68.7	66.6	64.7	71.2		48.2	14	Lrrn1b-Dr
15	79.3	79.3	79.3	78.5	78.9	79.7	78.5	79.7	77.8	81.8	80.2	75.2	81.8	66.8		15	Lrrn1b-Xl
	1	2	3	4	5	6	7	8	9	10	11	12	13	14	15		

C

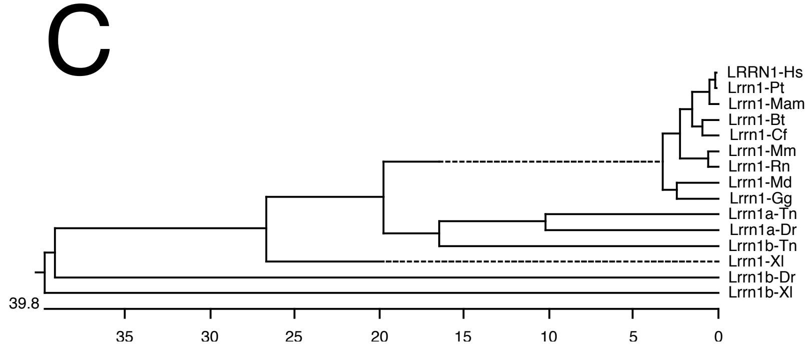

A) Clustal alignment of Lrrn1 proteins. Dashed lines represent gaps introduced by the alignment algorithm to produce an optimal alignment. Residues identical to Lrrn1-Gg are highlighted in black. Sequence positions are numbered on the left. Hs = *Homo sapiens* (ENSP00000314901), Pt = *Pan troglodytes* (ENSPTRG00000014566), Mam = *Macaca mulatta* (ENSMMUP00000001255), Bt = *Bos Taurus* (ENSBTAG00000001176), Cf = *Canis familiaris* (XP_541797), Mm = *Mus musculus* (ENSMUSP00000037096), Rn = *Rattus norvegicus* (XP_575630), Md = *Monodelphis domestica* (ENSMODP00000034019), Gg = *Gallus gallus*, Tn = *Tetradon nigroviridis* (Lrrn1a = GSTENT00014093001; Lrrn1b = GSTENT00027213001), Xl = *Xenopus laevis* (Lrrn1a = AAH59292, Xnlrr-1; Lrrn1b = ABC42025, XlNLRR-6), Dr = *Danio rerio* (Lrrn1a = XP_696951; Lrrn1b = XP_696356, zfLRRN). B) Table showing the percentage identity and percentage divergence between the aligned proteins. C) Cladogram showing phylogenetic relationship between Lrrn1 proteins. All panels were generated using the DNASTAR software module MegAlign
