# Supplementary material for: Analysis of Lrrn1 expression and its relationship to neuromeric boundaries during chick neural development
Source: Neural Dev. 2007 Oct 31;2:22. doi: 10.1186/1749-8104-2-22 (PMC2225406; doi:10.1186/1749-8104-2-22)
Supplement: Additional file 3 — Conserved motif in the intracellular domain of tartan, capricious and Lrrn1. Protein sequence alignment of conserved motif in the intracellular domain of tartan, capricious and Lrrn1. [file 1749-8104-2-22-S3.pdf]

Additional file 3: Conserved motif in the intracellular domain of tartan, capricious and Lrrn1

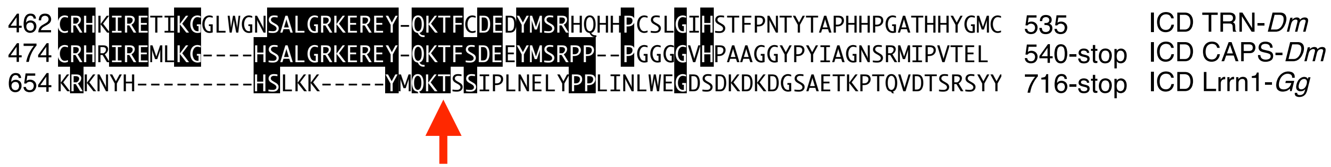

Clustal alignment of the intracellular domains (ICD) of tartan, capricious and chick Lrrn1. The entire intracellular domains of CAPS and Lrrn1 are shown. The ICD of TRN is truncated at residue 535. Dashed lines represent gaps introduced by the alignment algorithm to produce an optimal alignment. Residues identical across two sequences are highlighted in black. Sequence positions are numbered on the left and right. *Dm* = *Drosophila melanogaster (*TRN =NP_524055; CAPS = NP_729880, Gg = *Gallus gallus.*). Red arrow indicates the position of a hypomorphic allele of CAPS (T501I) within a short motif (QKT) conserved between all three proteins.

Alignment was generated using the DNASTAR software module MegAlign
