# Supplementary material for: Analysis of Lrrn1 expression and its relationship to neuromeric boundaries during chick neural development
Source: Neural Dev. 2007 Oct 31;2:22. doi: 10.1186/1749-8104-2-22 (PMC2225406; doi:10.1186/1749-8104-2-22)
Supplement: Additional file 2 — Comparison of chick and zebrafish Lrrn1 proteins. Protein sequence alignment of chick and zebrafish Lrrn1 proteins. [file 1749-8104-2-22-S2.pdf]

Additional file 2: Comparison of chick and zebrafish Lrrn1 proteins

A

```
1   MAKIRLVLTV-CQVVLELLTNSLTESSVQSNECPQLCVCEIRPWFTPQSTYREATTVDCNDLRLTKIPSNLSSDTQVLLLQSNNIAKTTDELQLLFNLTELDFSQNNFTSIRDVGLSNLT Lrrn1-Gg
1   MARGTFFLVVEGQICFILLLAVLGLSSVHSTECPQLCVCEIRPWFTPQSTYREATTVDCNDLHLTRIPGNLSADTQVLLLQSNYIARTSEELEQLLNLTELDLSQNNFSNIRDIGLTNMS Lrrn1a-Dr
1   MERTAISTSLLT-VCLVL---AVCRCSLAVPFCPAQCVCETRPWYTPQSVYHQAKTVDCNELHLSRIPWNISVDTQVLLLQSNNISRGTSQLQSLVNLTELDLSQNHFTQIHDVGLNNLT Lrrn1b-Dr

120 QLTTLHLEENQIMEMTDYCLQDLCNLQELYINHNQISSISANAFSGLKNLLRLHLNSNKLKVIDSRWFDSTPNLEILMIGENPVIGILDMNFKPLSNLRSLVLAGMYLTDIPGNALVGLD Lrrn1-Gg
121 QLTTLHLEENQIVEMPDFSLQDLTNLQELYINHNQISSIAPNAFAGLRNLLRLHLNSNRLKAIDSRWFESTPNLEILMIGENPVVGILDLNFKPLTNLRSLVLAGMELTDIPGNAFVGLD Lrrn1a-Dr
117 QLVTLYLEENQIKELPDMCLKDLVSLEELYINHNQISSIGPNAFSGWGNLLRLHLNSNKLVAIDSHWFESLPNLEILMIGENPILGLQDMNFHPLTKLHSLVLAGMGLREIPEGAFQGLE Lrrn1b-Dr

240 SLESLSFYDNKLVKVPQLALEKVPNLKFLDLNKNPIHKIQEGDFRNMLRLKELGINNMGELVSVDRYALDNLPELTKLEATNNPKLSYIHRLAFRNVPALESLMLNNNALNAVYQKTVES Lrrn1-Gg
241 NLESLSFYDNKLVRVPQTALQKLPNLKFLDLNKNPIHKIQEGDFKNMLRLKELGINNMGELVSIDRFALDNLPELTKLEATNNPKFSYVSRLAFRDLPALESLMLNNNALNSLYQATADS Lrrn1a-Dr
237 YLESLSFFDNKLTAVPKKALRVLPSLKFLDLNKNPIVRIQEGDFQDFPHLEELSLNNMEELVAVERGSFSNLPQMAKLELYNNPHLFFIDRAAFLKMRCLRTLLIHNNDLTLLPHEIVSA Lrrn1b-Dr

360 LPNLREISIHSNPLRCDCVIHW--INSNKTNIRFMEPLSMFCAMPPEYRGQQVKEVLI---QDSNEQCLPMISHETFPNHLNLDIGMTVFLDCRAMAEPEPEIYWVTPLGNKVTVESLS- Lrrn1-Gg
361 LPNLREISIHSNPLRCDCVIQW--MSSNKTTIRFMEPLSMFCTMPIEVRGQRVRDVFS---REPSGQCLPMISHDTFPNHLNLDIGMTVDLDCRAMAEPEPEIYWVTPSGNKVMMDTVS- Lrrn1a-Dr
357 FPNLDEISLHSNPLRCDCLNNWGPVLGNQSSLKVLEPQITLCASPQHLVGQALQDVVSASWNGASNTCLPLISQHAFPPQLNVTLGQLLTLDCWAVADPAPQFYWVTPTGDKVTSEAVSP Lrrn1b-Dr

474 ---------DKYKLSSEGTLEISNIQIEDSGRYTCVAQNIEGADTRVATIRVNG-------------TLLDGTQVLKIFVKQAESHSILVSWKVNSNVMTSNL-----KWSSATMKIDNP Lrrn1-Gg
475 ---------DKYQLNSAGTLRISYIQVDDSGHYTCVAQNTEGADTRVTAIRVNG-------------TLLDSTQLMKIYVKHTESHSILVSWKVNSNVMTSNL-----KWSSATMKIDNP Lrrn1a-Dr
477 SSNEGGGTPKKHRMQDQGALEIPHIEPEDTGLYTCVAWNAEGADTRSVSVYVDKRSWNGAHHFGGHHGVVNTTGSLVILAKTVHAQSVVLEWKMHPYAIPSNHEAGQPKWLSATVKIDNP Lrrn1b-Dr

567 HITYTARVPVDVHEYNLTHLQPSTDYEVCLTVSNIHQQTQKSCVNVTTKNAAFALDISDQETSTALAAVMGSMFAVISLASISVYIAKRFKRKNYHHSLKKYMQKTSSIPLNELYPPLIN Lrrn1-Gg
568 HITYTAKVPVDVHEYNLTHLQPATEYEVCLSVSNIHQQTQKSCVNVTTKHAMFAVEISEQGTNTALAAVMGTILAIISLGSITLYIAKRWKRKNYHHSLKKYMQKTSSIPLNELYPPLIN Lrrn1a-Dr
597 QISYTAMVPVDVQEYNLTHLLPSTEYQVCLTMAGT-EQTQHSCINVTTKEASFAVEMVAQPTNVALAAVMGSMFAICIMALLVFYMGRRMKQKSCHHSLKKYMQHTTSIPLNELYPPLIN Lrrn1b-Dr

687 LWEGDSDKDKDGSAETKPTQVDTSRSYYMW                                                                                           Lrrn1-Gg
688 LWEADSEKDKDGSSDNKQTQVDTTRSYYMW                                                                                           Lrrn1a-Dr
716 LWENETEKEKEGAVDPQNSQIDTSKTY-MW                                                                                           Lrrn1b-Dr
```

B

Percent Identity (upper right) / Divergence (lower left)

	1	2	3		
1		80.2	50.8	1	Lrrn1-Gg
2	22.5		49.9	2	Lrrn1a-Dr
3	64.7	66.5		3	Lrrn1b-Dr
	1	2	3		

C

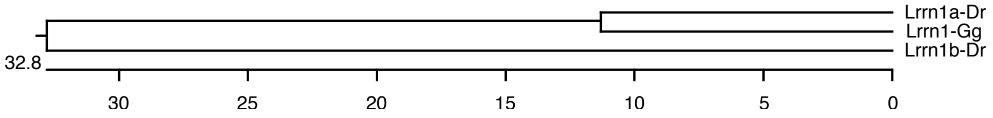

A) Clustal alignment of chick and zebrafish Lrrn1 proteins. Dashed lines represent gaps introduced by the alignment algorithm to produce an optimal alignment. Residues identical to Lrrn1-Gg are highlighted in black. Sequence positions are numbered on the left. Lrrn1-Gg = chick (*Gallus gallus*); Lrrn1a-Dr = zebrafish (*Danio rerio*, XP_696951); Lrrn1b-Dr (zfNLRR (XP_696356). B) Table showing the percentage identity and percentage difference between the aligned proteins. Lrrn1-Gg and Lrrn1a-Dr share 80% amino acid identity whereas Lrrn1b-Dr is divergent and shares approximately 50% amino acid identity with Lrrn1/1a. C) Cladogram showing phylogenetic relationship between Lrrn1 proteins. All panels were generated using the DNASTAR software module MegAlign
